# The Role of Optical Coherence Tomography in the Early Detection of Chiasmal Compression: Hypophyseal Adenoma Presenting With Bitemporal Nerve Fiber Layer Thinning

**DOI:** 10.7759/cureus.55371

**Published:** 2024-03-02

**Authors:** Joobin Khadamy

**Affiliations:** 1 Ophthalmology, University Hospital of Umeå, Umeå, SWE

**Keywords:** cabergoline treatment, mri, macroadenoma, asymmetrical visual field defects, chiasmal damage, biatemporal or binasal nerve fiber layer thinning, visual field defects, oct, optical coherence tomography, hypophyseal adenoma

## Abstract

Hypophyseal adenomas can present with or without minimal visual disturbances. We present a case of a 40-year-old male with a hypophyseal adenoma, highlighting bitemporal peripapillary retinal nerve fiber layer (NFL) thinning on optical coherence tomography (OCT) as a major sign of chiasmal damage despite minimal asymmetrical nonspecific changes detected on initial visual field testing. The bitemporal NFL thinning prompted further evaluation with MRI, which confirmed the presence of a macroadenoma of the hypophysis. Despite the large adenoma, treatment with cabergoline led to regression, and the patient's visual field improved. This case underscores the importance of OCT in detecting subtle structural changes associated with pituitary tumors, as it can facilitate early diagnosis and prompt intervention for optimal visual outcomes.

## Introduction

Hypophyseal adenomas, particularly macroadenomas, constitute a substantial portion of pituitary tumors and they frequently result in visual disturbances due to their proximity to crucial optic structures. These tumors are estimated to comprise between 35 and 65% of all pituitary adenomas, underlining their clinical significance. While hypophyseal macroadenomas can affect individuals of any gender, asymptomatic cases are more prevalent in men, emphasizing the importance of proactive screening and early detection efforts [1].

Visual disturbances, encompassing reduced visual acuity, visual field defects, and diplopia, are the most common presenting symptoms in these patients, with visual field defects often the earliest indicators of chiasmal compression. These defects can vary from subtle asymmetrical findings to more classical bitemporal hemianopia, showcasing the heterogeneous clinical presentations of these tumors. Other common signs and symptoms of hypophyseal adenomas include headaches, hormonal imbalances resulting in symptoms such as fatigue and irregular menstruation, and compression-related symptoms like pituitary gland dysfunction leading to excessive thirst and urination, as well as nausea and vomiting [1].

Optical coherence tomography (OCT) has emerged as a valuable diagnostic modality for assessing hypophyseal macroadenomas, particularly in detecting bitemporal peripapillary nerve fiber layer (NFL) thinning indicative of chiasmal compression. Such thinning, identified on OCT, holds prognostic significance, providing valuable insights into potential visual outcomes. We discuss a case of a patient presenting with minimal monocular symptoms rather than the classical bitemporal defect, highlighting the importance of comprehensive visual field assessment and the potential diagnostic and prognostic value of OCT in managing hypophyseal adenomas [2].

## Case presentation

The patient was a 40-year-old man who sought medical attention due to visual disturbances. Upon evaluation, he reported experiencing a left-sided central visual field defect in his left eye following the resolution of a large central chalazion on the left upper eyelid. Notably, the patient did not report any associated headaches or other neurological symptoms. His visual acuity was minimally affected in the left eye (0.9) while remaining normal in the right eye (1.0). Color vision and eye motilities were within normal limits, but a relative afferent pupillary defect was noted in the left eye.

**Figure 1:**
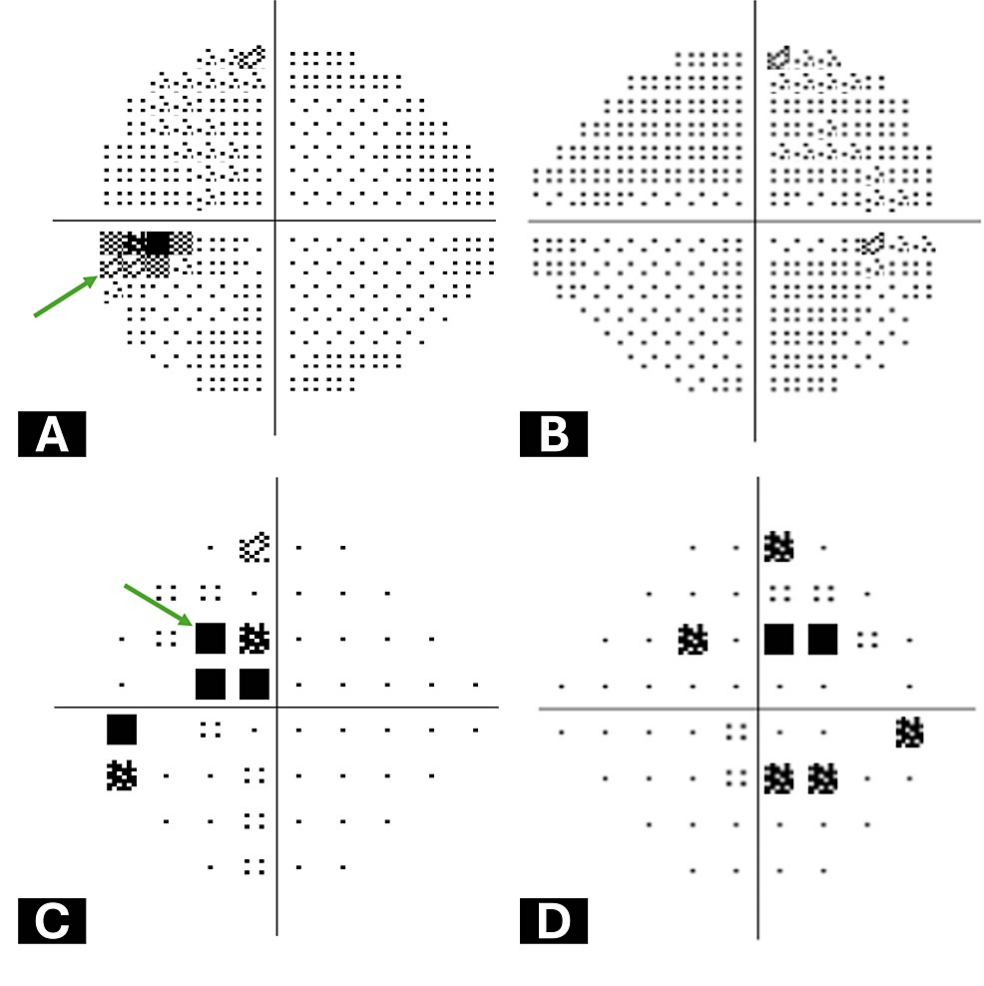
Initial Humphrey central 24-2 visual field testing results A: Grayscale map of the left eye demonstrating enlargement of the blind spot (Green arrow). B: Grayscale map of the right eye displaying diffuse changes without a specific pattern. C: Pattern deviation map of the left eye revealing a temporal cecocentral defect (Green arrow). D: Pattern deviation map of the right eye illustrating defects in the temporal and nasal parts of the visual fields with no specific pattern

Initial assessment via 24-2 Humphrey visual field testing revealed minimal changes, predominantly enlargement of the blind spot (Figure 1A), and a temporal cecocentral scotoma in the left eye (Figure 1C).

**Figure 2:**
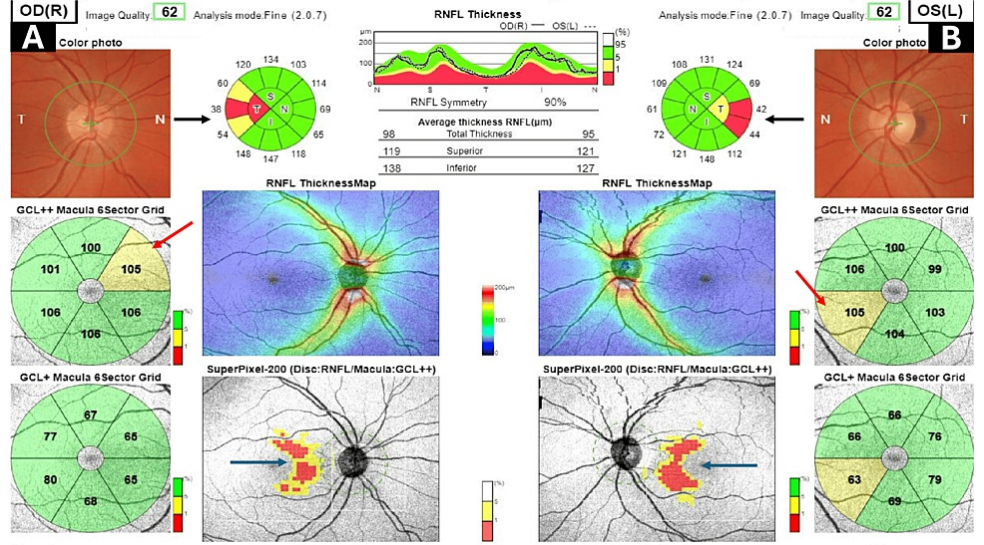
A 3D widefield OCT glaucoma report of the right and left eyes A and B: RNFL thickness map showing bitemporal thinning (black arrows) in the right and left eyes. GCL++ macula 6 sector grid displayed supranasal thinning in the right eye (A) and inferonasal thinning (red arrows) in the left eye (B). SuperPixel-200 (disc: RNFL/macula: GCL++) demonstrating bitemporal nerve fiber layer thinning (dark blue arrows) OCT: optical coherence tomography; RNFL: retinal nerve fiber layer

Subsequent evaluation with OCT revealed bitemporal NFL thinning, particularly evident in the 3D widefield glaucoma scan (Figure 2).

**Figure 3:**
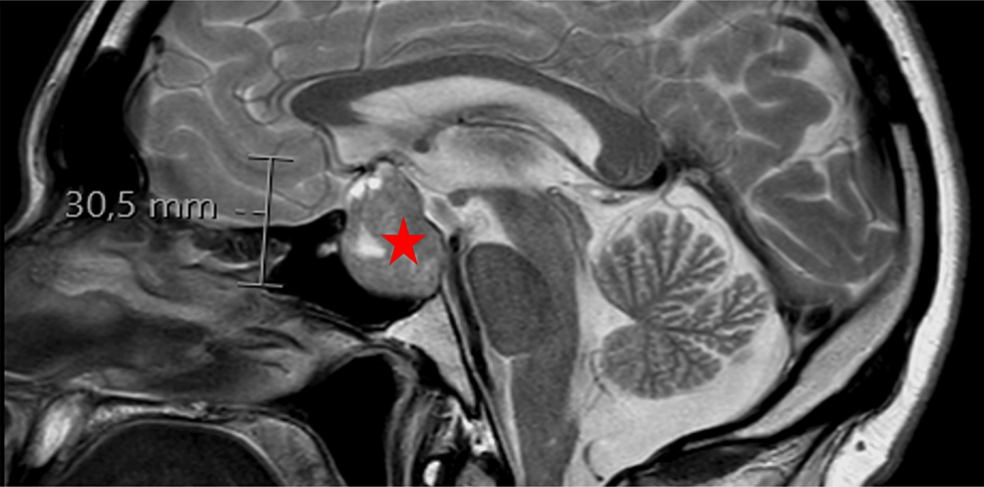
MRI showing a hypophyseal macroadenoma T2W MRI sagittal section displaying a 30.5 mm hypophyseal macroadenoma (red star) with suprasellar extension grade 3 according to SIPAP grading. Signs of erosion are evident in the posterior wall of the sella, and the chiasma is elevated by the adenoma MRI: magnetic resonance imaging

Further imaging with MRI confirmed the presence of a 30.5 mm macroadenoma of the hypophysis, characterized by suprasellar extension grade 3 according to SIPAP grading (Figure 3). Parasellar extension was grade 1 according to Knosp grading. Signs of erosion were evident in the posterior wall of the sella, and the chiasma was lifted by the adenoma.

**Figure 4:**
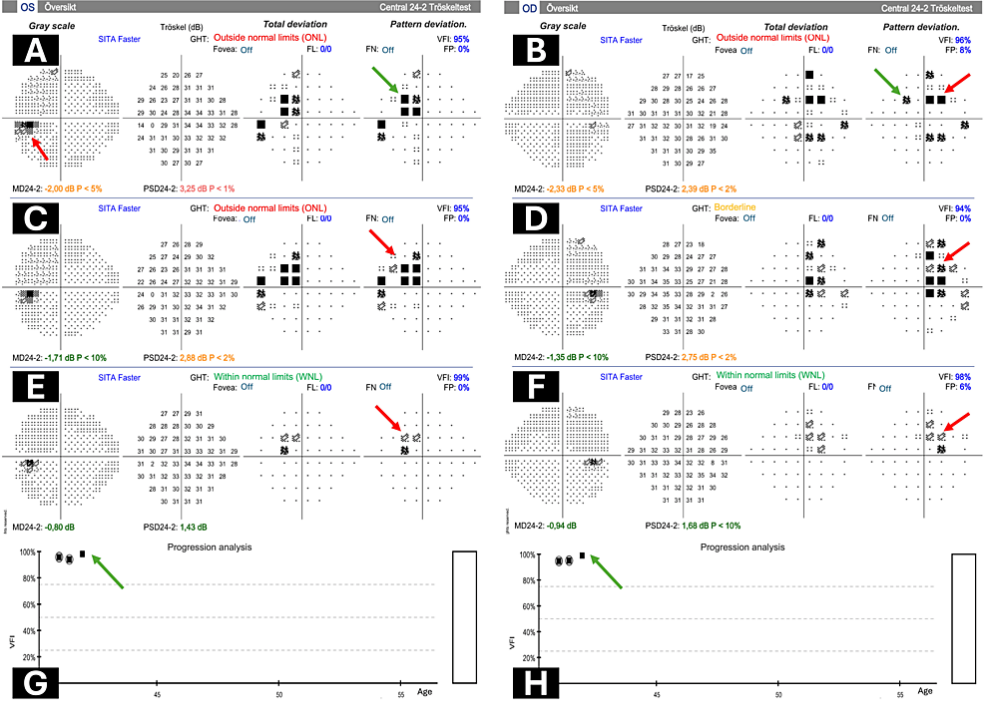
Visual field overview and global progression analysis report during follow-up A-F: The overview visual field report. A: The grayscale map of the left eye demonstrates enlargement of the blind spot (red arrow). Pattern deviation map of the left eye revealing a temporal cecocentral defect (green arrow). B: Grayscale map of the right eye displaying diffuse changes without a specific pattern but both nasal (green arrow) and temporal defects (red arrow) on pattern deviation map. C and D: Six-month follow-up visual field of the left and right eye demonstrating progression and more obvious bitemporal visual field scotoma (red arrows). E and F: Follow-up at one-year showing improvements and near normal indices after cabergoline treatment, although there is still some trace of cecocentral bitemporal scotoma (red arrows). G and H: Global progression analysis demonstrating improvement over the one-year follow-up period (green arrows)

Hypophyseal hormone analysis revealed hyperprolactinemia (>200 ng/ml). Following a discussion at the tumor board, the patient was treated with cabergoline. Follow-up visual field examinations demonstrated initial progression and subsequent improvement after cabergoline treatment, indicating a positive response to treatment (Figure 4). These findings underscore the dynamic nature of visual field changes in response to therapeutic interventions and highlight the importance of longitudinal monitoring in the management of hypophyseal adenomas.

## Discussion

Hypophyseal adenomas, particularly macroadenomas, are commonly associated with visual disturbances due to their proximity to critical optic structures. Visual field defects are among the earliest signs of chiasmal compression, often presenting as asymmetrical findings or classic bitemporal hemianopia [3]. Table 1 summarizes the visual field deficits associated with hypophyseal adenomas.

**Table 1:** Various visual field deficits associated with hypophyseal adenomas RAPD: relative afferent pupillary defect

Visual field deficit	Description	Mechanism	Compression side
Monocular visual field deficits or junctional scotoma of Traquair	Temporal (more common) or nasal monocular hemianopsia	Postfixed chiasm or anterior asymmetrical tumor spread	Ipsilateral optic nerve before chiasm
Junctional scotoma	Ipsilateral central scotoma and contralateral superior temporal visual field defect	Postfixed chiasm or anterior asymmetrical tumor spread	Ipsilateral optic nerve and Wilbrand's knee of the chiasm
Bitemporal hemianopsia (most common)	Progress from superior to complete bitemporal hemianopsia (can be asymmetrical initially)	Normal chiasm compressed by tumor from below	Chiasm
Cecocentral scotoma	Paracentral bitemporal hemianopsia	Prefixed chiasm or posterior tumor spread	Macular fibers on the posterior of the chiasm
Homonymous hemianopsia	Contralateral visual field loss, RAPD, and ''band'' or "bow-tie" optic atrophy	Prefixed chiasm or posterior asymmetrical tumor spread	Optic tract

In our case, initial Humphrey visual field testing revealed minimal changes, emphasizing the importance of comprehensive assessment techniques. The pattern deviation map proved particularly informative, revealing a temporal cecocentral defect in the left eye. These findings highlight the necessity of evaluating both pattern deviation and grayscale maps to detect subtle abnormalities indicative of chiasmal compression. Additionally, it is important to consider the potential role of the neurological hemifield test (NHT) in evaluating pathologies affecting the visual pathways. While the glaucoma hemifield test (GHT) is commonly utilized to identify global visual field abnormalities, it may not always detect focal defects. In our case, despite the normalization of GHT, residual visual losses were evident in the pattern deviation map, indicating localized defects. This suggests that incorporating the NHT alongside GHT could offer a more comprehensive assessment of visual field deficits in neurologic cases. However, it is essential to note that NHT was not assessed in the current case, emphasizing the necessity for further exploration of its potential utility in such contexts [4].

OCT has revolutionized the diagnosis and management of hypophyseal adenomas by enabling the detection of early structural changes, particularly bitemporal peripapillary NFL thinning. Our case demonstrated bitemporal peripapillary NFL thinning on OCT, prompting further evaluation with MRI, which confirmed the presence of a macroadenoma of the hypophysis. This highlights the promising diagnostic and prognostic value of OCT in detecting subtle structural abnormalities associated with pituitary tumors [5]. Furthermore, OCT enables noninvasive and objective monitoring of treatment response, aiding in the assessment of therapeutic efficacy and predicting visual outcomes [6].

The multidisciplinary approach to patient care was pivotal in the management of the presented case. Collaboration between ophthalmologists, neurosurgeons, and endocrinologists facilitated comprehensive evaluation, accurate diagnosis, and timely intervention. This underscores the importance of a multidisciplinary team in optimizing treatment outcomes for patients with complex neuro-ophthalmic conditions.

In terms of treatment, the patient was initiated on cabergoline therapy following a discussion at the tumor board. Cabergoline, a dopamine agonist, has been shown to effectively reduce tumor size and alleviate symptoms associated with pituitary adenomas, including visual field deficits [1]. Long-term follow-up demonstrated initial progression of visual field defects, followed by subsequent improvement after treatment with cabergoline. This highlights the dynamic nature of visual field changes in response to therapeutic interventions and emphasizes the importance of close monitoring in the management of hypophyseal adenomas.

## Conclusions

This case report underscores the diagnostic utility of advanced imaging modalities, including wide-field OCT scans, in detecting bitemporal peripapillary NFL thinning associated with hypophyseal adenomas. Multidisciplinary collaboration played a pivotal role in facilitating accurate diagnosis, timely intervention, and optimized treatment outcomes. Further research is warranted to elucidate the role of OCT in guiding treatment decisions and predicting visual outcomes in patients with pituitary tumors. Early detection and intervention remain paramount in preserving visual function and improving quality of life in patients with hypophyseal adenomas.
